# Review of Molecular Technologies for Investigating Canine Cancer

**DOI:** 10.3390/ani14050769

**Published:** 2024-02-29

**Authors:** Alexandra Kehl, Heike Aupperle-Lellbach, Simone de Brot, Louise van der Weyden

**Affiliations:** 1 Laboklin GmbH & Co. KG, Steubenstr. 4, 97688 Bad Kissingen, Germany; kehl@laboklin.com (A.K.); aupperle@laboklin.com (H.A.-L.); 2 School of Medicine, Institute of Pathology, Technical University of Munich, Trogerstr. 18, 81675 München, Germany; 3 Institute of Animal Pathology, COMPATH, University of Bern, 3012 Bern, Switzerland; simone.debrot@unibe.ch; 4 Wellcome Sanger Institute, Wellcome Genome Campus, Hinxton, Cambridge CB10 1SA, UK

**Keywords:** canine, dog, DNA, RNA, miRNA, epigenetics, genome, transcriptome, sequence, PCR

## Abstract

**Simple Summary:**

Genetic molecular testing is starting to become part of standard clinical practice for dogs with cancer as it can assist veterinarians with diagnosis, provide information about prognosis and aid with selection of therapeutic options. In this review, we consider the different body fluids and tissues from which tumour cells, DNA, RNA and the relevant proteins can be isolated, and what methods are currently used for characterising the molecular profile of cancer in dogs. We also consider new methods that are currently being developed and look promising. The aim is to provide an overview of molecular technologies for veterinarians without previous experience in molecular biology.

**Abstract:**

Genetic molecular testing is starting to gain traction as part of standard clinical practice for dogs with cancer due to its multi-faceted benefits, such as potentially being able to provide diagnostic, prognostic and/or therapeutic information. However, the benefits and ultimate success of genomic analysis in the clinical setting are reliant on the robustness of the tools used to generate the results, which continually expand as new technologies are developed. To this end, we review the different materials from which tumour cells, DNA, RNA and the relevant proteins can be isolated and what methods are available for interrogating their molecular profile, including analysis of the genetic alterations (both somatic and germline), transcriptional changes and epigenetic modifications (including DNA methylation/acetylation and microRNAs). We also look to the future and the tools that are currently being developed, such as using artificial intelligence (AI) to identify genetic mutations from histomorphological criteria. In summary, we find that the molecular genetic characterisation of canine neoplasms has made a promising start. As we understand more of the genetics underlying these tumours and more targeted therapies become available, it will no doubt become a mainstay in the delivery of precision veterinary care to dogs with cancer.

## 1. Introduction

Molecular profiling in cancer concerns the assessment of alterations that are found in tumour cells relative to normal cells. Specifically, this involves the characterisation of genomic alterations (changes in the DNA), transcriptomic alterations (changes in RNA/gene expression) and epigenomic alterations (reversible modifications that affect gene expression without altering the DNA sequence).

### 1.1. The Genome and Genetic Alterations

**Figure 1:**
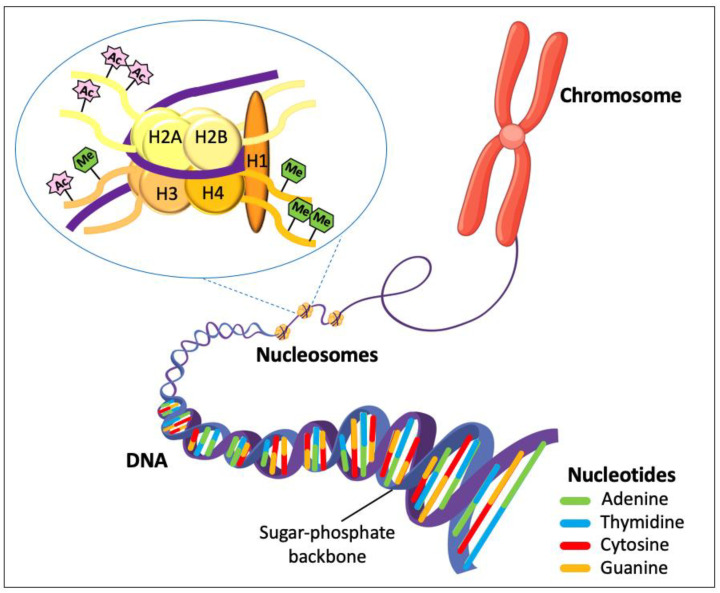
Schematic of the DNA structure. The nitrogenous bases of the two separate strands of DNA are bound together by hydrogen bonds between the complementary bases (A–T and G–C) and, together with the sugar-phosphate backbone, form a double-stranded helix. A nucleosome is the basic unit of DNA packaging and consists of a segment of DNA wound around a core octamer of 8 histone proteins. The ‘tails’ of the histone proteins can be modified by a variety of epigenetic enzymes such as methyltransferases (leaving a ‘Me’ mark) and acetyltransferases (leaving an ‘Ac’ mark) at specific sites, which can lead to the activation or inhibition of gene expression, depending on the situation/context. Parts of this figure have been designed using images from Freepik.com (accessed on 16 December 2023).

The ‘genome’ is the complete set of DNA molecules within the organism (or population of cells), which, in canines, would be the DNA present in the 39 pairs of chromosomes in the nucleus and the relatively small amount of DNA present in the mitochondria (mtDNA). DNA consists of monomeric units called nucleotides, the basic structure of which is a nitrogenous base, a pentose sugar (deoxyribose), and a phosphate group. The four nitrogenous bases consist of the purine bases, adenine (A) and guanine (G), and the pyrimidine bases, cytosine (C) and thymine (T), as shown in Figure 1.

**Figure 2:**
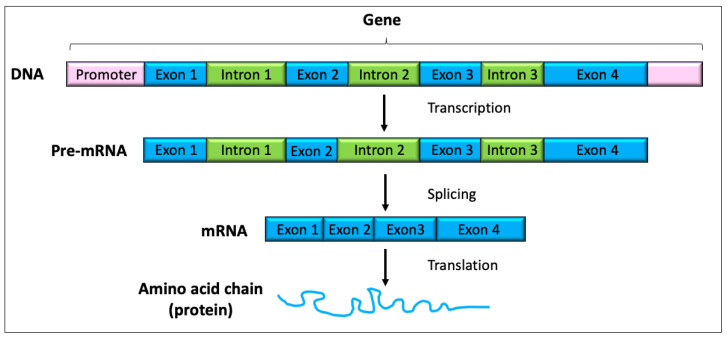
Genes are composed of promoters, followed by alternating regions of exons and introns. During transcription, the entire gene is copied into pre-messenger RNA (pre-mRNA), which includes all the exonic and intronic sequences. During splicing, the intronic sequences are removed, and the exonic sequences are joined together to form a contiguous ‘mature’ mRNA sequence. The mRNA is then transported out of the nucleus to the ribosomes in the cytoplasm, where it can be used to direct protein synthesis, together with transfer RNA (tRNA), in a process known as translation.

DNA has to be highly condensed to fit into the nucleus; therefore, histone proteins are used to condense the DNA into chromatin. The basic chromatin structure is the nucleosome, which consists of DNA wrapped around the histone octamer (comprising two copies each of the histone proteins H2A, H2B, H3 and H4) and the histone H1 protein that binds to linker DNA between the nucleosomes (Figure 1). Genes are the parts of the genome that encode the information for making a protein and are typically composed of exons and introns (Figure 2). Exons are regions of DNA that are transcribed to RNA and are retained after introns are spliced out (see Figure 2 and Section 1.2 below).

**Figure 3:**
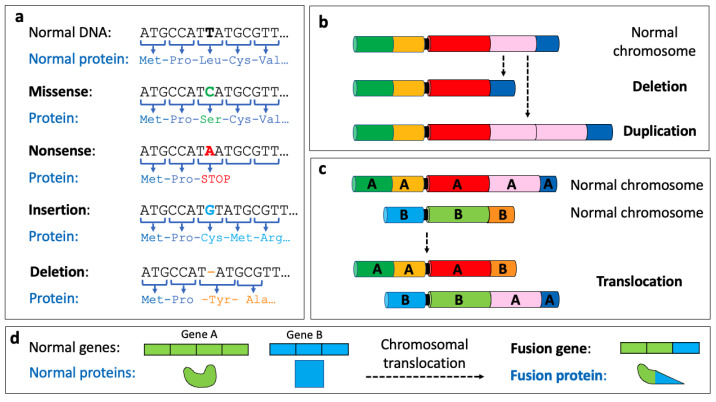
Types of genetic alterations. (**a**) Single-nucleotide variants are created by point mutations, which affect a single nucleotide. These can be missense mutations, nonsense mutations or insertions/deletions (collectively called ‘indels’). The effect of the DNA alteration on the resulting amino acid sequence is shown using the 3-letter code for amino acids (below the arrow). Amino acids not in dark blue indicate an alteration from the original protein sequence. Indels can result in frameshift mutations, i.e., a shift in the 3-base pair reading frame that alters the downstream amino acid sequence. (**b**) Structural variants (SVs) include deletions (loss of regions of the chromosome) or duplications (amplification/gain of regions of the chromosome) and result in copy number alterations. (**c**) Translocations are SVs that do not result in a change of copy number; however, as in (**d**), they can result in the formation of a fusion gene (where parts of two different genes are spliced together).

The DNA can possess many different types of genetic alterations, both somatic (i.e., those present only in the tumour cell) and germline (i.e., those present in every cell in the body and are passed onto the offspring), which are typically divided into small-scale and large-scale variants. Small-scale variants include single-nucleotide variants (SNVs, also known as ‘point mutations’) such as missense mutations, nonsense mutations and insertions/deletions (‘indels’) (Figure 3a).

Small-scale variants can also involve multiple nucleotides, such as indels. Indels can result in ‘frameshift mutations’ that can produce incomplete or incorrect proteins (Figure 3a). Large-scale variants, also known as ‘structural variants’ (SVs), can involve losses or gains of large parts of a chromosome, a chromosome arm or a whole chromosome, and these are referred to as copy number alterations (CNAs; Figure 3b). Other SVs include chromosomal translocations (where a portion of one chromosome breaks away and adds on to another chromosome; see Figure 3c). These chromosomal rearrangements can produce ‘fusion genes’, a hybrid gene formed of parts of two previously independent genes, and these can play a role in driving tumourigenesis (Figure 3d).

### 1.2. The Transcriptome and Alterations in Gene Expression

Transcription is the process by which the information in a strand of DNA is copied into a new molecule of single-stranded immature messenger RNA (pre-mRNA). The pre-mRNA molecules then undergo ‘splicing’, in which the non-coding introns are cut out, leaving only the coding exons. Splicing produces a mature mRNA molecule that is then transported to the ribosomes in the cytoplasm, where it is used to direct protein synthesis in a process known as translation. The ‘transcriptome’ is the complete set of protein-coding mRNA transcripts and the non-coding RNA transcripts (such as tRNA and rRNA) within the organism or population of cells.

Gene expression is frequently reregulated/altered in tumour cells. A type of gene frequently altered in cancers is a proto-oncogene, which typically promotes cell growth. When mutated, proto-oncogenes become oncogenes; they are permanently activated and can result in tumourigenesis, due to uncontrolled cell growth. Another type of gene that is frequently altered in cancers is a tumour suppressor gene (TSG), which typically functions to prevent uncontrolled cell growth. When mutated, they lose their ability to work normally, so cell growth goes unchecked.

### 1.3. The Epigenome and Epigenetic Alterations

Epigenetics is the study of heritable changes in gene expression that do not alter the DNA sequence. The epigenome is the set of chemical modifications (‘marks’) to the DNA and DNA-associated proteins in the genome, which can alter gene expression by determining which genes are turned ‘on’ or ‘off’ in cells at specific times. The DNA-associated proteins are the histone proteins, which control the architecture of the chromatin, the positions of the nucleosomes and, thus, the accessibility of the DNA for transcription of the genes in that region (Figure 1). While these chemical modifications can occur as part of natural processes, they can also occur in response to environmental exposure or diseases such as cancer. There are two kinds of marks, specifically, DNA methylation and histone modification. DNA methylation is carried out by DNA methyltransferase enzymes and results in a methyl group being added to the cytosine within a CpG dinucleotide, forming 5-methylcytosine (5-meC). ‘CpG regions’ (or ‘CpG islands’) are short stretches of DNA with an unusually high GC content and a higher frequency of CpG dinucleotides compared to the rest of the genome. They are usually located in the promoter (5′ regulatory) region of genes, and the methylation of this region is typically associated with gene silencing (or switching ‘off’ the gene). In contrast, DNA demethylation is typically associated with gene activation (or switching ‘on’ the gene). Histone modification is when the histone proteins, not the DNA, undergo modifications such as methylation, acetylation, phosphorylation, or ubiquitination. Histone methylation can lead to the activation or inhibition of gene expression, depending on the situation/context (Figure 1). Histone acetylation is associated with the opening of the chromatin and the activation of gene expression, whereas de-acetylation leads to chromatin compression and the silencing/inactivation of gene transcription. Another form of epigenetic regulation of gene expression is carried out by non-coding RNAs (ncRNAs), which are RNA molecules that are not translated into proteins. These include long non-coding RNAs (lncRNAs), microRNAs (miRNAs), piwi-interacting RNAs (piRNAs), and endogenous small interfering RNAs (siRNAs). These ncRNAs can interact with DNA methyltransferases or histone-modifying complexes and, in doing so, regulate gene expression. In addition, some ncRNAs are targets of epigenetic alterations themselves.

Aberrant epigenetic regulation contributes to cancer development by altering gene expression. For example, tumour cells may show silencing of key TSGs through the methylation of their promoter region or deacetylation of the histone proteins surrounding that region. In addition, some ncRNAs are highly expressed in cancer cells and promote cancer development, while others can act as tumour suppressors.

### 1.4. Oncogenomics

Oncogenomics is the study of cancer-associated genes and focuses on the genomic, transcriptomic and epigenetic alterations that occur in cancer. A thorough understanding of the oncogenome has deeply influenced the management of human cancer patients, from aiding in diagnosis and prognosis to heralding the era of precision therapeutic strategies. As cancer is a significant cause of morbidity and mortality in domestic dogs, comprehensive canine oncogenomic studies are critical to paving the way for achieving a similar level of patient care for our canine pets by providing the groundwork for the identification of new diagnostic markers, prognostic markers for tumour classification and precision/targeted therapies. Indeed, several recent studies in the canine oncology field have demonstrated the strong clinical impact that oncogenomics can have. For example, genomic analysis of 191 dogs with splenic haemangiosarcoma identified both the somatic mutations and germline variants associated with clinical variables, including breed and overall survival [1], while genomic analysis of 828 dogs across 53 tumour types found that almost 90% of the cases exhibited mutations with diagnostic, prognostic or therapeutic implications [2]. In another study of 127 dogs for which genomic analysis and clinical outcomes were available, mutations in 6 genes were significantly associated with shorter progression-free survival (PFS; *CND1*, *CCND3*, *SMARCB1*, *FANCG*, *CDKN2A/B*, and *MSH6*), and dogs that received targeted treatment before first progression showed significantly longer PFS than those that did not, including those dogs that received genomically informed targeted treatment [3]. A study of the prognostic effect of genomic alterations in 1108 dogs found prognostic concordance between humans and canines in several key cancer-associated genes, including *TP53* and *PIK3CA*, and several human-targeted treatments were associated with a positive prognosis when used to treat canine tumours with specific genomic alterations [4].

However, a comprehensive understanding of these genomic alterations is only possible with the cutting-edge molecular profiling technologies that have been initially developed for use in human oncology and are gradually finding their way into routine use in veterinary oncology. To this end, we review the ‘where to look?’ aspects of molecular profiling, in terms of which methods can be used to sample tumour cells in dogs, and the ‘how to look?’ aspects of molecular profiling, in terms of what tools are currently available to interrogate the canine oncogenome.

## 2. Methods for Sampling Tumour Cells in Dogs

Molecular genomic analyses can be performed using various body materials [5], which are discussed in detail below.

### 2.1. Tissue Samples

The most traditional source for obtaining tumour cells for genetic characterisation is fine needle aspirates (FNA; cytology) or tissue samples (histology). The tissue samples may be collected for diagnostic purposes by an excisional biopsy or during surgical removal of the tumour mass. Even though the number of tumour cells obtained by FNA is lower than in a biopsy, they are still sufficient to be used for genomic investigations. Typically, this would involve more targeted approaches such as PCR to detect mutations in a specific gene (technique detailed in Section 3.1 below) rather than next-generation sequencing (NGS; technique described in Section 3.5 below). For example, mutations in exon 11 of *KIT* (also known as *c-KIT*) have been examined by PCR on FNAs of canine mast cell tumours [6].

**Figure 4:**
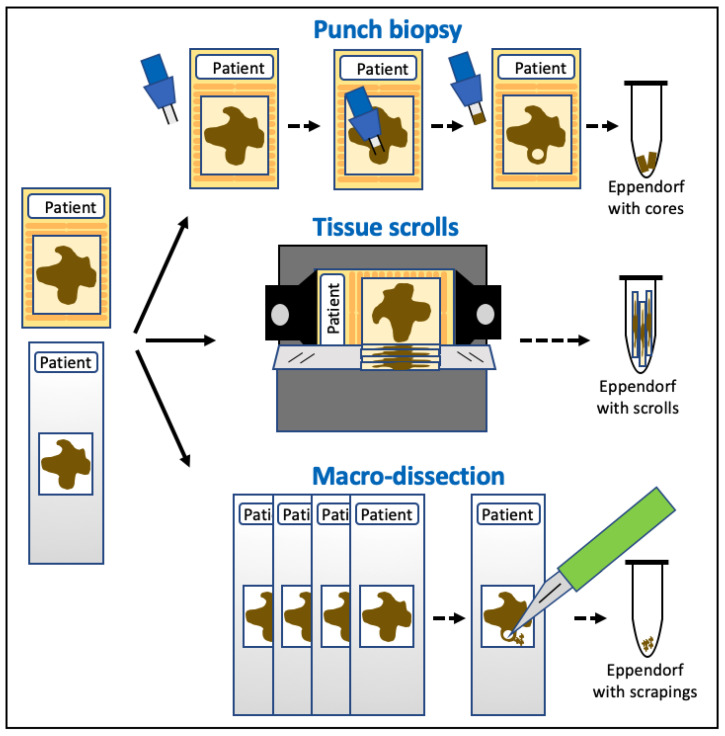
Methods for collecting tumour samples from formalin-fixed, paraffin-embedded (FFPE) blocks. From the FFPE block, a thin section is cut and placed onto a glass slide for staining with haematoxylin and eosin to allow histological evaluation. Once the sample has been determined to be suitable, there are three options for sampling the tissue in the block (represented by the black arrows). (i) Biopsy punches (‘cores’) can be taken from specific areas in the block. (ii) Curled-up tissue sections (‘scrolls’) can be collected from the microtome to cut the sections. (iii) Unstained tissue sections can be collected onto slides and then the appropriate area is macro-dissected by ‘scraping’ with a scalpel. In all cases, the tissue samples are collected into Eppendorf tubes for DNA and/or RNA extraction.

In general, the preference is for fresh or ‘fresh-frozen’ (FF) tissue (where the tissue is snap-frozen in liquid nitrogen at the point of collection and then stored at −80 °C); however, in contrast to human medicine, the storage of fresh-frozen tissue in a biobank is not routine in veterinary medicine. Therefore, a widely used alternative is formalin-fixed paraffin-embedded tissue (FFPE). The sampling of FFPE tissues to collect the tumour cells for molecular analysis can be achieved in various ways, depending on the size of the tumour sample and/or the presence of normal tissue, as shown in Figure 4.

However, there are important limitations to using nucleic acids (DNA or RNA) extracted from FFPE samples for molecular analysis [7,8]. Formalin is an aqueous formaldehyde solution and can have a range of detrimental effects on nucleic acids. It can cause cross-linking, fragmentation/degradation and chemical modification of the nucleic acid to introduce artefacts, all of which interfere with the various techniques used in molecular studies and/or can lead to false signals. These issues are of particular concern for next-generation sequencing-based techniques, and so will be discussed further in Section 3.5 below. Nevertheless, FFPE-extracted DNA and RNA are routinely used in genomic medicine in humans [7,9] as there are steps that can be put in place to manage these known issues and minimise them where possible. For example, to try and obtain higher-quality DNA/RNA from the samples, regard must be given to sample ascertainment, based on aspects of the fixation process such as the pH and concentration of the formalin, fixation temperature and time, thickness of the tissue, and specimen storage conditions [8,10]. The nucleic acid extraction process should also be taken into consideration, with the establishment of evidence-based best practices for DNA extraction from FFPE to help ensure the accuracy and reproducibility of molecular analyses performed on FFPE-extracted nucleic acids [11,12,13]. The use of canine FFPE tumour samples in the available molecular technologies is detailed in the relevant parts of Section 3 below.

### 2.2. Liquid Biopsy

Other sources of tumour cells for molecular investigation are the ‘fluids’ of the body, such as the blood, urine, saliva, effusions and cerebrospinal fluid, as well as collection method-induced fluids, such as bronchial lavage and peritoneal washes. Sampling of these fluids is grouped under the collective term ‘liquid biopsy’, and there are a range of parameters that can be used for genomic investigation.

#### 2.2.1. Circulating Tumour Cells

During cancer progression and metastasis, tumour cells can detach from the primary tumour and enter into the bloodstream (circulating tumour cells; CTCs), whereby they can travel to distant organs and extravasate (disseminated tumour cells; DTCs), followed by colonisation of the organ to become clinically relevant metastases. In veterinary medicine, only a few studies have performed molecular analyses of CTC in dogs to date; they have all been conducted from a diagnostic point of view rather than via genomic characterisation of the CTCs (i.e., detecting CTCs is an indicator of the presence of a tumour and/or disease progression). Typically, CTCs have been identified by detecting the presence of specific mRNAs in the blood by means of RT-PCR (a technique detailed in Section 3.1.2). For example, by considering the mRNAs present in canine mammary carcinoma cell lines (CMM26 and CMM115) and tumours, but not in a healthy dog’s peripheral blood leukocytes (PBLs), one group determined that *CRYAB* mRNA was a highly specific and moderately sensitive marker for the detection of CTCs in dogs with metastatic mammary carcinoma [14,15,16].

#### 2.2.2. Circulating Cell-Free DNA

Cell-free DNA (cfDNA) is released from the cells into the blood (although it is also found in a variety of other body fluids). It is present in healthy patients, arising through the physiological processes of apoptosis, necrosis and active secretion. However, in certain physiological conditions (such as pregnancy [17]) or certain pathological conditions (such as cancer [18]), the related/affected tissues can release additional DNA fragments into the blood or other body fluids. In cancer patients, the part of the cfDNA derived from tumour cells is the circulating tumour DNA (ctDNA), which carries tumour-related genetic and epigenetic alterations. In veterinary medicine, studies have shown that dogs with mammary carcinoma [19] and lymphoid neoplasia [20] have higher plasma cfDNA concentrations relative to those of controls. More recently, the presence of somatic alterations in cfDNA has been detected. For example, using ddPCR (a technique detailed in Section 3.1.4), a study identified somatic *PTPN11* E76K or G503V mutations in the tissue of histiocytic sarcoma tumours from 23/45 dogs, with the mutations also being found in the plasma of 21/23 of the same dogs [21]. Similarly, ddPCR found copy number alterations (amplifications) of *MDM2* in oral malignant melanoma tumours from 3/10 dogs, with the amplifications also detected in the plasma of 1/3 of the same dogs [21]. Recently, a commercial multi-cancer early detection (MCED) screening test has become available for canines in the USA that provides a ‘Cancer Signal Detected/Cancer Signal Not Detected’ result based on the NGS identification of proprietary cancer-associated genomic alterations in cfDNA from the blood (OncoK9^®^ from PetDx). However, it is essential to understand that both the tumour size and its type influence the probability of a positive cancer signal [22,23].

In addition to cfDNA in the blood, cfDNA in the urine of dogs has also been used for genomic characterisation. For example, NGS of FFPE tissue samples has demonstrated the presence of the somatic *BRAF* p.V595E mutation in a significant proportion of canine urinary bladder urothelial carcinomas (UC) [24], while a study using NGS identified the presence of the tumour genotype (*BRAF* p.V595E) in 9/9 of the matched urine sediment samples from dogs with urinary bladder UC [25]. Similarly, using PCR and Sanger sequencing (a technique detailed in Section 3.4), the results of *BRAF* p.V595E analysis in dogs with or without urinary bladder UC were identical between the FFPE tissue, cytological smear and urine sample [26].

#### 2.2.3. Nucleosomes

In the cell, DNA exists in the form of chromatin, a complex structure composed of DNA and histone proteins packaged together in the form of nucleosomes. Nucleosomes consist of a segment of ~150 base pairs of DNA, wound around 8 histone proteins (specifically, 2 copies each of the proteins, H2A, H2B, H3 and H4), and are the predominant form of cfDNA in the plasma (cfNucleosomes) [27]. Histones are modified by the covalent attachment of various chemical groups, resulting in epigenetic patterns unique to each tissue. Importantly, cfNucleosomes retain some of these post-translational modifications (PTMs), and the quantification of nucleosomes and specific PTMs shows potential as cancer biomarkers for breast and colorectal cancer in humans [28,29]. The ‘Nu. Q™ H3.1 Assay’ is designed for the detection of levels of histone H3.1-containing cfNucleosomes in human serum and has also been used to show that serum from canines with lymphoma contains a 7-fold-increased nucleosome level in their plasma relative to healthy control dogs [30]. Using this assay, a recent study quantified and characterised nucleosome concentrations in both healthy dogs (*n* = 134) and those dogs with various cancer types (*n* = 528) and found that tumours of haematopoietic origin were most likely to result in increased nucleosome concentrations in the plasma, whereas local tumours such as soft tissue sarcomas were the least likely to have this result [31]. However, it is important to note that the levels of nucleosomes can only be used for cancer detection in an otherwise clinically healthy dog, as other studies have shown that concentrations of nucleosomes are significantly higher in dogs under certain conditions relative to healthy controls, such as dogs with sepsis, with acute gastroenteropathies or following trauma [32,33,34].

#### 2.2.4. MicroRNAs

MicroRNAs (miRNA, miR) are single-stranded, non-coding RNA molecules transcribed from DNA that bind target mRNA to negatively regulate their expression (and, thus, prevent protein production). To protect them from degradation (by RNases), the release of miRNAs is broadly categorised into two different biochemical compositions, specifically, exosomal (extracellular vesicles) and non-exosomal. Although miRNAs play a vital role in regulating numerous homeostatic metabolic and cellular pathways, dysregulated exosomal and non-exosomal miRNA expression profiles have been found in most tumours [35]. Although miRNAs are seen as a potential diagnostic tool for cancer detection, their use in routine clinical practice in human medicine is still in its infancy [36,37,38], with issues related to identifying a tumour-specific miRNA signature due to the high variability and diversity of miRNAs present in both healthy individuals and those with disease (whether neoplastic or non-neoplastic in origin).

In canines, qRT-PCR (a technique detailed in Section 3.1.3) has been used to show significantly elevated levels of miR-214 and miR-126 in the plasma of canines with haemangiosarcoma [39] and osteosarcoma [40] and significantly higher levels of miR-20b, miR-148a-3p and miR-652 in the serum of dogs with gastrointestinal tumours (lymphomas and/or carcinomas) [41], relative to healthy dogs. Analysis of the serum of dogs by ddPCR found that circulating miR-19b and miR-125a were significantly elevated in dogs with mammary carcinoma relative to those without [42]. Analysis by RNAseq (a technique detailed in Section 3.5.2) found that circulating miR-18a levels were significantly higher in those samples with histologic evidence of lymphatic invasion [42]. Another study using qRT-PCR on serum from dogs with mammary tumours found that miR-21 overexpression and miR-29b downregulation were associated with clinical stages [43,44]. In lymphomas, analysis of serum by qRT-PCR found that miR-205, miR-222, and miR-20a were upregulated, miR-93 was downregulated and several other miRNAs were mis-regulated in specific subtypes of lymphomas [45]. Another study also used qRT-PCR analysis of serum and found that miR-223, miR-25 and mi-R92a were downregulated in the serum of dogs with lymphoma [46]. miRNA analysis has also been performed on urine from dogs, with RT-PCR determining that miRNA-16, 34a, 103b and 106b levels were elevated in dogs with urinary bladder UC [47,48].

## 3. Methods for the Genetic Characterisation of Tumour Cells in Dogs

### 3.1. Polymerase Chain Reaction (PCR)

#### 3.1.1. PCR Using DNA

**Figure 5:**
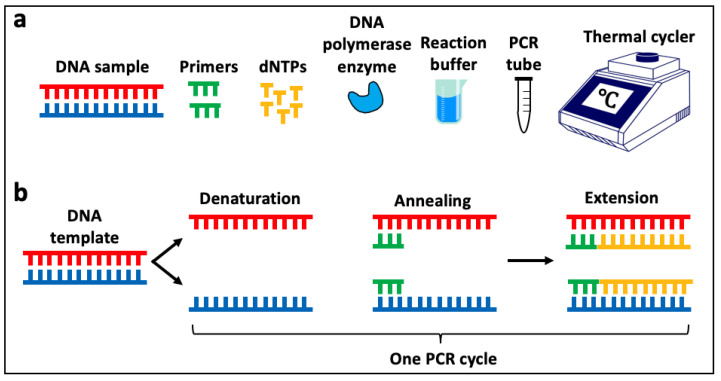
Polymerase chain reaction (PCR). (**a**) The key components of PCR are the DNA sample, primers, nucleotides (dNTPs), DNA polymerase enzyme and reaction buffer, which are all placed into a PCR tube and put inside a thermal cycler for the PCR reaction to occur. (**b**) The PCR reaction (one cycle) is a three-step process. The first is the denaturation step, performed at a high temperature (usually ~95 °C) to break apart the hydrogen bonds holding the two DNA strands together. The second is the annealing step, performed at a lower temperature (typically 55–65 °C) to allow the primers to anneal (bind) to the DNA template at the complementary sequence. The third is the extension step, performed at the optimal temperature required by the particular DNA polymerase (typically 65–72 °C) to allow the polymerase to start synthesizing the new strand of DNA, commencing at the primer annealing region. After a certain period of time (determined by the rate of synthesis of the particular polymerase), the PCR solution is then heated to a high temperature so that the ‘denaturation-annealing-extension’ cycle can repeat. Some of the images in this figure were from Freepik.com (accessed on 16 December 2023).

PCR is a method used to amplify specific portions of DNA (target regions) from a small amount of DNA template. Specifically, it is used to generate millions of copies of the target region of DNA to increase its ability to be detected and analysed in downstream applications. A standard PCR requires a DNA polymerase enzyme (which catalyses the synthesis of DNA molecules from the nucleotides), nucleotides (ATP, TTP, GTP and CTP, collectively known as dNTPs), primers (short, single-stranded nucleic acid sequences that provide a starting point for the DNA polymerase enzyme), reaction buffer, and the DNA template (Figure 5a).

The three main steps in PCR are denaturation, annealing and extension, and one round of each of these steps, known as a ‘cycle’, results in two copies of the targeted portion of the original template (a strand of DNA), as shown in Figure 5b. After many cycles, millions of copies of the targeted region will have been generated (known as ‘PCR products’). The PCR technique is widely used in canine cancer studies, typically with modifications to the protocol to allow for quantification of the PCR product (as detailed in Section 3.1.3 and Section 3.1.4 below) or identification of the sequence of the PCR product (as described in Section 3.4 below).

#### 3.1.2. PCR Using RNA

PCR using RNA as the starting material (template) is termed reverse transcription PCR (RT-PCR), as the RNA is first transcribed into complementary DNA (cDNA) by a reverse transcriptase enzyme, and then the standard PCR procedure is used to amplify the cDNA. RT-PCR is used mainly for gene expression analysis. The RT-PCR technique is typically used with modifications to the protocol to allow quantification of the PCR product and, thus, quantification of the RNA transcripts present in the original sample (as detailed in Section 3.1.3 below) or identification of the sequence of the PCR products (as detailed in Section 3.4 below).

#### 3.1.3. Quantitative PCR (qPCR)

qPCR (also known as ‘real-time’ PCR) allows sensitive quantification of the target DNA. As in standard PCR, the DNA is amplified by cycles of denaturation, annealing and elongation; however, by adding probes with fluorescent dyes (fluorophores) to the PCR reaction, it is possible to measure the amount of PCR product being produced as the PCR progresses (i.e., in real-time). The fluorescence intensity changes at each cycle correlates to the quantity of target DNA (template) that was put into the reaction, and comparison to an internal reference sample allows for the quantification. RT-qPCR is for the analysis of RNA or miRNA and functions in the same way as qPCR, with the exception that RNA is the nucleic acid starting template; as such, the addition of an initial ‘reverse transcriptase’ step is needed to convert the RNA to cDNA, which is then used in the qPCR reaction.

Recent examples of RT-PCR being used to quantitatively examine gene expression levels in canine tumours include determining that *ATOX1* is over-expressed in canine osteosarcoma, similar to that found in mouse and human osteosarcoma [49], and *MYC* is upregulated in canine Meibomian gland neoplasms relative to normal glands [50]. RT-qPCR has also been used to validate array gene expression data. For example, based on results obtained from gene expression profile microarray data comparing metastatic and non-metastatic canine mammary tumours [51], RT-qPCR confirmed the ‘metastatic gene signature’ of eight genes by determining significant differences in mRNA expression levels between the metastatic and non-metastatic tumours [51]. RT-qPCR was also used to determine that *Sfrp1* mRNA levels were significantly increased in canine mammary tumours without lymph node metastases [52]. Similarly, RT-qPCR has been used to validate gene expression changes detected by RNAseq, with one study finding that overall, the expression trends between RNAseq and RT-qPCR were highly comparable when comparing mRNA levels of cancer-associated stromal cells between non-metastatic and metastatic canine mammary carcinomas [53]. In contrast, another study found that whilst some RT-qPCR results were concordant with the statistically significant expression changes in miRNA levels seen between canine normal/minimally inflamed duodenal tissue, severe lymphoplasmacytic inflammatory bowel disease and T-cell lymphoma groups identified by RNAseq, others were discordant [54].

#### 3.1.4. Digital PCR (dPCR)

**Figure 6:**
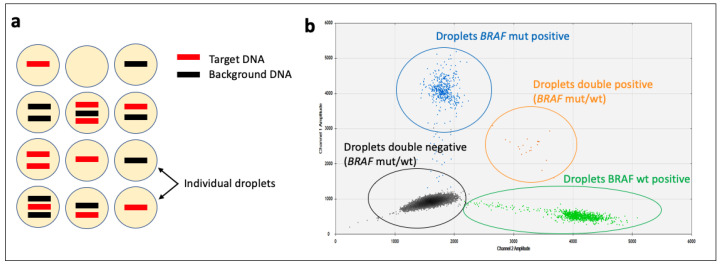
Droplet digital PCR (ddPCR). (**a**) The sample DNA is partitioned into thousands of individual droplets, which results in minimal amounts of DNA for amplification within each droplet. (**b**) ddPCR results for *BRAF* p.V595E in urine from a dog with urinary bladder urothelial carcinoma. ‘*BRAF* mut’ indicates the presence of the *BRAF* p.V595E mutation in the DNA and ‘*BRAF* wt’ indicates the presence of the wild-type (‘unmutated’) allele in the DNA sample.

Although qPCR and RT-qPCR are considered quantitative, the quantification provided is only a ‘relative’ quantification, as the results are reported with respect to the expression of a control (a ‘housekeeping gene’), or a standard curve may be produced through serial dilutions. Digital PCR (dPCR) is a PCR-based technique that enables the ‘absolute’ quantification of target regions of DNA by extreme limiting dilution and partitioning of the samples into many tens of thousands of compartments/units (or ‘droplets’, when using ‘droplet dPCR’ or ddPCR) that contain either DNA, with or without the portion of interest, or no DNA (Figure 6a). As each unit contains all the components of a PCR reaction, if the target region of interest is present, then the amplification step will yield a fluorescent signal. If it is not present, no fluorescent signal is produced (hence the term ‘digital’, as the quantitation is binary, i.e., a signal or no signal). If the number of partitioning units is known, then the initial amount of target DNA molecules that were present in the sample can be quantitated [55]. In addition, simultaneous analysis of several targets in a single sample is possible by multiplexing.

dPCR does not need an external standard, thus making it more precise and reproducible than qPCR [55], and it is typically used when high sensitivity is needed; the test is able to detect target molecules at a much lower frequency than qPCR (it can detect mutant DNA in a background of 100,000-fold excess of wild-type DNA) [56]. As such, it is often used on liquid biopsy samples, where the amount of ctDNA is only a small fraction of the cfDNA. For example, ddPCR has been used to identify the *BRAF* p.V595E mutation in urine samples from dogs with urothelial and prostatic carcinoma [57] (Figure 6b). It has also been used on FFPE tissues, such as for identifying the *BRAF* p.V595E mutation in canine digital melanoma [58] or a two-base (‘AT’) insertion mutation in *TP53* in canine histiocytic sarcoma [59]. In addition, dPCR can also measure copy number alterations (CNAs; known as copy number variations (CNVs) for germline samples) [56], with changes of less than a 1.2-fold difference able to be detected [60]. For example, in the identification of patterns of CNAs in specific regions of canine chromosomes 8, 13, 19 and 36, dPCR could be used to reliably distinguish between FFPE samples of canine neoplastic and non-neoplastic bladder tissues, and CNAs were also able to be detected by dPCR in more than half of the urine samples from dogs with urinary bladder UC [57]. In addition, dPCR analysis for identifying the presence of four key CNAs has been used to predict aggressive tumour phenotypes in canine mast cell tumours [61]. Finally, dPCR can also be performed in an RT-PCR context, as demonstrated by its recent use to determine *CK19* mRNA expression levels in canine peripheral blood and freshly collected mammary tissue samples [62] and miR-18b, -20b, -126, -192, -194, and -214 levels in FFPE canine intestinal B-cell or T-cell lymphomas and carcinomas [63].

### 3.2. DNA Microarray

Microarrays are collections of single-stranded DNA probes, complementary to the target sequence of interest, which are bound in defined positions to a solid surface (such as a glass slide or beads). Nucleic acid fragments from the sample of interest are labelled with fluorescent dyes (either by PCR or RT-PCR) and then hybridised with the microarray, followed by washing to remove any non-specific hybrids that may have formed. A laser then excites the attached fluorescent dyes to produce light that is detected by a scanner, which then generates a digital image from the excited microarray, after which specialized software transforms the image of each spot into a numerical reading. The amount of hybridisation (fluorescence) detected for a specific probe is directly proportional to the number of nucleic acid fragments present in the sample. In two-colour microarrays, two different biological samples (typically, a test sample and a control sample) are each labelled with different fluorescent dyes, usually Cyanine 3 (Cy3; green) and Cyanine 5 (Cy5; red). Equal amounts of nucleic acid are then simultaneously hybridised to the same microarray chip (‘competitive hybridisation’) and separate fluorescence measurements are made for each dye. The reading then represents the abundance of each gene in one sample relative to the other.

#### 3.2.1. Gene Expression Arrays

DNA microarray technologies were initially designed as a way to measure the expression levels of thousands of RNA transcripts within the genome in a single experiment. A common type of experiment is to extract RNA from the tumour and normal tissues of the same dog, label them with different fluorescent dyes, simultaneously hybridise them to the same microarray, and then use the relative fluorescence intensities produced at each spot to indicate differences in the amount of each transcript between the two samples. For example, one study performed comparative cDNA microarray expression profiling of canine mammary tumour tissues and healthy mammary gland tissues and identified 1700 and 1287 significantly differentially expressed genes (DEGs) in the malignant and benign tissues, respectively, relative to the healthy tissue [64]. The DEGs were functionally annotated using the Ingenuity Systems Pathway Analysis (IPA) tool to help explore the pathways and interaction networks associated with the development and pathogenesis of canine mammary tumours [64]. Another study performed gene expression profiling of 20,000 genes to identify those associated with the survival times of dogs with osteosarcoma and found 51 DEGs, with the overexpressed genes identified in the ‘short survival time’ cohort being associated with possible roles in proliferation, drug resistance or metastasis [65]. Similarly, one study used gene expression microarrays to determine the differences in expression profiles between metastatic and non-metastatic canine mammary tumours [51].

#### 3.2.2. Array-Based Comparative Genomic Hybridization (aCGH)

aCGH is a type of microarray developed for detecting copy number changes in the genome and quantitatively comparing the fluorescence signal strength between the test DNA (such as tumour DNA) and the reference/control DNA (such as healthy tissue DNA from the same dog) for each spot on the array. The relative fluorescence intensities produced indicate copy number changes at each spot. Early studies used small-scale microarrays [66]; however, now genome-wide aCGH is commonplace. For example, a study characterising FFPE canine mast cell tumours with *KIT* mutations showed genome-wide aberrant copy number profiles, with frequent copy number alterations (CNAs) involving genes in the p53 and RB pathways, in stark contrast to tumours with wildtype *KIT*, which showed very limited numbers of CNAs [61]. Another study found that canine oral melanoma showed recurrent chromosomal gains (regions of amplifications) involving chromosomes CFA 10, 13 and 30 and recurrent chromosomal losses (regions of deletions) involving chromosomes CFA 10, 11, 22 and 30, with the most frequently involved genes in those regions being *MAPK*- and *PI3K*-related genes [67]. Similarly, analysis of canine androgen receptor-negative prostate cancer samples found recurrent regions of chromosomal losses spanning well-known TSGs, including *ATM*, *BRCA1*, *CDH1*, *MEN1* and *TP53* [68].

#### 3.2.3. Microarray-Based DNA Methylation Profiling

Methylation microarrays have been developed to allow for an assessment of the methylation status of individual CpG loci across the genome. In human studies, a popular method is to ‘genotype’ the C/T conversion that results from bisulphite treatment of the DNA, with the proportion of DNA methylation at a particular CpG site then being ascertained by taking the ratio of the methylated (C) to unmethylated (T) signals. A study investigating the epigenome of canine diffuse large B-cell lymphoma (DLBCL) designed a bespoke CpG microarray platform, in which oligo-probes were designed against >40,000 CpG regions and coding sequences (CDS) distributed across the entire dog genome, and DNA methylation was measured by 2-colour competitive hybridisation between the methylated ‘enriched’ fraction and the ‘not-enriched’ portions of each DNA sample [69]. Analysis of the samples (*n* = 37 DLBCLs and *n* = 7 control lymph nodes) identified > 1194 loci showing differential methylation levels between the tumours and the controls, with members of the Polycomb Group genes being mostly affected, and also allowed identification of DLBCL subgroups, with the accumulation of aberrant methylation resulting in more aggressive behaviour on the part of the tumour [69].

### 3.3. Quantitative Nuclease Protection Assay (qNPA)

Nuclease protection assays (NPA) are highly sensitive techniques designed for the detection and quantitation of mRNA. In particular, the qNPA is able to reliably analyse expression levels in fragmented mRNAs from small amounts of tissue and, thus, is highly suited for use with FFPE samples [70]. The assay has been shown to give comparable results to those obtained by gene expression profiling using RT-qPCR or DNA microarrays [70,71,72]. A key to the success of this method is its ability to measure mRNA without having to extract it from the tissue, generate cDNA (see Section 3.1.2 above) or perform gene amplification, as is necessary for RT-qPCR or microarray procedures [70,72]. The FFPE tissue is sectioned, permeabilised and denatured (by being heated to a high temperature) and hybridised with specific cDNA sequences of interest (termed ‘nuclease protection probes’). Thus, the qNPA procedure results in a 1:1 replacement of each mRNA transcript with its cDNA sequence/probe. The tissue sections are then treated with nuclease to destroy any probes that have not hybridised to a mRNA transcript, followed by alkaline hydrolysis to release the mRNA-bound probes from the tissue, leaving intact probes in concentrations that are directly proportional to the amounts of specific mRNA originally present in the tissue. The freed mRNA transcript probe is then transferred to an ‘ArrayPlate’, which uses an oligonucleotide array and a sandwich hybridization linker to capture and label the probes for chemiluminescent or fluorescent detection and subsequent quantification [72].

A pilot study to assess the analytical performance of qNPA to simultaneously measure the RNA expression of multiple genes in archived FFPE canine tumour tissues used 40 canine tumours with a 96-well ArrayPlate that was built using oligonucleotide probes for 30 canine-specific genes (in addition to housekeeping genes and positive/negative controls) [73]. The assay was found to be linear with decreasing sample input and was also reproducible within and between ArrayPlates and different laboratories. Once validated, 70 FFPE canine tumours were analysed for differences in gene expression, and gene signature patterns across tumour types were identified that provided insights into the molecular mechanisms of canine cancers, particularly related to the expression levels of *MDM2* and *E2F1*; *MDM2* was commonly upregulated across tumour types, particularly melanomas, while *E2F1* was significantly differently expressed between B- and T-cell lymphomas and different grades of sarcoma [73].

### 3.4. Sanger Sequencing

**Figure 7:**
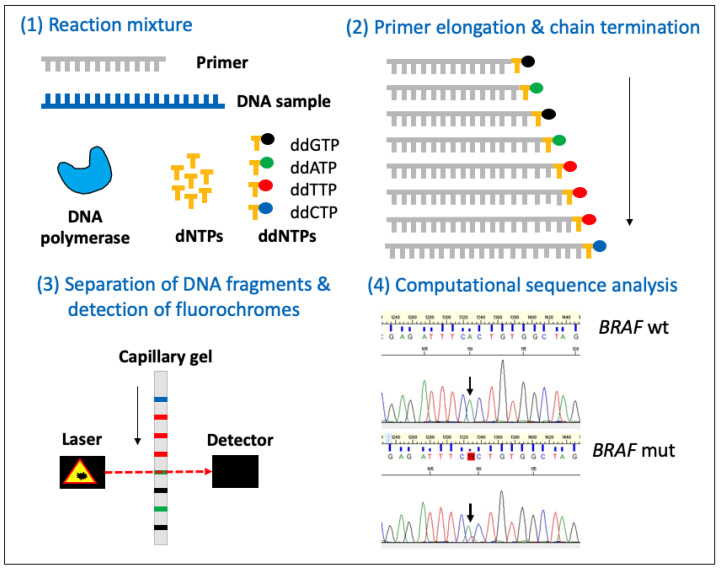
The Sanger (chain-termination) method for DNA sequencing. The Sanger sequencing method requires single-stranded DNA (to act as a template), a primer, a DNA polymerase, deoxynucleotide triphosphates (dNTPs) and modified dNTPs (ddNTPs), which together make up the PCR reaction. The ddNTPs are ‘chain-terminating nucleotides’ that terminate DNA strand elongation. They are also fluorescently labelled to allow their detection once the DNA has been size-separated by capillary gel electrophoresis. They are detected by lasers (red arrow), which then feed the information to a computer. Computational analysis is then required to generate the sequence (as shown on the two chromatograms; sequencing of DNA from canines with urothelial carcinoma—one that was wild-type (wt) for *BRAF* (upper panel) and one that was mutant (mut) for *BRAF* (p.V595E; lower panel), as shown by the base/nucleotide that is present where the arrow is pointing).

Sanger sequencing, also known as the ‘chain termination method’, is a method for determining the nucleotide sequence of DNA. Sanger sequencing involves PCR (as detailed above) but, in addition to dNTPs, it also uses fluorescent, chain-terminating modified nucleotides (ddNTPs, with each of the four nucleotides having a unique fluorescent label), resulting in ‘fragments’ of the DNA region of interest, each terminated at a random length by a 5′-ddNTP. The DNA fragments are then separated by capillary gel electrophoresis based on their size (they appear as a ‘band’ on the gel). The gel is subsequently analysed by a computer to ‘read’ each band, using fluorescence to identify the identity of each 5′-ddNTP, with the different ‘reads’ being compiled to generate the sequence of the region of DNA that was amplified (Figure 7).

To date, Sanger sequencing has been used in many hundreds of studies to determine the DNA sequence of individual genes or the particular exons of genes from canine tumours. Some examples include Sanger sequencing of RT-PCR and PCR products to identify the ITDs in exon 11 of *KIT* from RNAlater^®^-preserved canine cutaneous mast cell tumour tissue [74] and from canine digital mast cell tumour FFPE tissue [75]. Similarly, Sanger sequencing has been used to identify the *BRAF* p.V595E mutation in PCR products from FFPE canine urothelial and prostatic carcinoma tissue [57] (Figure 7) and canine papillary oral squamous cell carcinoma (OSCC) [76].

### 3.5. Next-Generation Sequencing (NGS)

NGS, also known as high-throughput sequencing, massively parallel sequencing or deep sequencing, is an all-encompassing term that describes a number of modern sequencing technologies that allow the sequencing of DNA and RNA at much greater speed and capacity than conventional Sanger sequencing, providing data that are orders of magnitude more, and at a much lower relative cost. As such, it has revolutionised the field of genomics and, in particular, has boosted our understanding of canine oncogenomics due to its ability to simultaneously interrogate millions of targets. There are a variety of NGS techniques currently available, each investigating different aspects of the genetic composition of an organism, along with a variety of computational analysis programs, each aimed at identifying specific genetic alterations. They can broadly be divided into three groups, based on the genomic modifications/variations they are designed to detect, specifically: (i) genetic variations—those in the DNA, (ii) transcriptional variations—those in the RNA, and (iii) epigenetic variations—those affecting DNA methylation, histone modifications and microRNAs.

#### 3.5.1. NGS for Detecting Variations in the DNA

**Figure 8:**
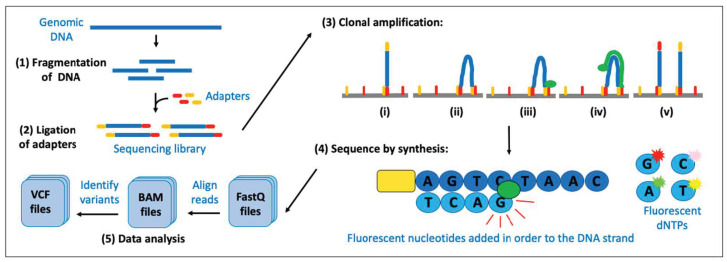
Workflow for next-generation sequencing (NGS), using whole genome sequencing as an example of an NGS workflow. Step 1: The genomic DNA is fragmented. Step 2: A ‘library’ is then created by ligating adapters (yellow and red) to the fragmented DNA. Step 3: The library is then loaded into the flow cell of a sequencing machine and ‘clonal amplification’ can occur: (i) the DNA attaches to oligonucleotides in the flow cell via complementary sequences within its adaptor; (ii) the strand bends over and attaches to a second oligonucleotide forming a ‘bridge’; (iii) a polymerase enzyme (green) binds to the sequence in the adapter and (iv) synthesizes a complementary (reverse) strand; (v) the two strands release and straighten. For further amplification, each new strand can form a new bridge, and after several iterations of cycles, the result is a cluster of DNA clones (both forward and reverse strands). At the end of this process, all the reverse strands are washed from the flow cell. Step 4: ‘Sequence by synthesis’ then occurs, whereby a primer attaches to the primer binding site in the adapter of the forward strand and a polymerase (green) adds a fluorescently tagged dNTP to the DNA strand (each dNTP has a different fluorophore). Only one dNTP is able to be added per round, due to the fluorophore functioning as a blocking group; thus, once the fluorophore colour is recorded, the fluorophore is washed away and another dNTP is added over the flow cell and the process is repeated. Step 5: After sequencing is complete, computational analysis of the data is required to first filter them for quality and amplicon size (data are stored as a FastQ file), align them to a reference genome (data are stored as a binary alignment mapping or ‘BAM’ file), and then perform variant calling (data are stored as a variant calling format or ‘VCF’ file). Some images are from Freepik.com (accessed on 16 December 2023).

There are three commonly adopted methods for using NGS to detect variations in the DNA, specifically, whole genome sequencing (WGS), whole exome sequencing (WES) and targeted sequencing (TGS). WGS involves sequencing all the nucleotides in the genome, including chromosomal DNA and mitochondrial DNA, and, as such, can determine variations in any part of the genome. WES involves sequencing the entire coding region (i.e., all the exons) of the genome. TGS involves sequencing a selected (i.e., targeted) portion of the genome that is specified by the user as to their genes/regions of interest. A typical NGS experiment shares the same workflow, regardless of the specific technologies that may be used, and can be divided into four steps (Figure 8).

After extraction of the DNA from the tissue sample, the first step in the NGS process is known as ‘library preparation’. This firstly requires processing the genomic DNA into relatively short fragments (typically 100–800 bp), which can be achieved with a variety of techniques, and then ligating (attaching) ‘adaptor sequences’ to the DNA to form the ‘library’ for that sample. Adapters contain three different parts:

(1) A sequence that is complementary to the solid support. The solid support comprises the oligonucleotides that are covalently attached to the surface of the flow cell of the sequencing machine and are required for the ‘cluster amplification’ step later in the procedure.
(2) A ‘barcode’ sequence. This is a short unique tag. All fragments of DNA from one sample (library) have adapters containing the same tag, to allow for multiple libraries to be mixed together and sequenced at the same time (known as ‘multiplexing’ or ‘pooling’).
(3) A binding site for the sequencing primer. This is required for the ‘sequencing’ step later in the procedure.

At this point in the process, if there is <500 ng of DNA, an amplification step is typically performed. The whole library of sequences is amplified by PCR using primers that recognise part of the adapter sequence, often for only 6–10 cycles. For WES or TGS, an additional step known as ‘target enrichment’ is subsequently performed, whereby the samples are hybridised with biotinylated complementary probe sequences (‘baits’). This step allows for a ‘pulldown’ of the portion of the genome that is of interest (i.e., the probes are designed against all the coding regions of the genome for WES baits and the targeted regions of the genome for TGS baits). By collecting/capturing the regions of the genome that have bound to the baits, washing away the unbound fraction and digesting away the biotinylated probes, the sample has then been enriched for the regions of interest and is ready for the second step of the NGS protocol.

While all NGS platforms perform parallel sequencing of millions of small DNA fragments, several different technologies are available, and the choice of which technology to use depends on the specific needs of the study, such as the desired read length, throughput and accuracy (Table 1). The most widely used platform is Illumina.

Using Illumina technology, the libraries are then loaded onto the sequencing machine, where the second step in the NGS process can occur: clonal amplification (also known as ‘cluster amplification’). This amplifies a single molecule on the surface of the flow cell in a clonal manner to increase the number of molecules being sequenced (Figure 8). WGS can be performed with or without clonal amplification; however, WES and TGS analyses always use this step to ensure that there is a sufficient library to be sequenced. The libraries are then sequenced using the ‘sequencing by synthesis’ method, which involves the sequencing machine ‘reading’ the individual bases (nucleotides) as they are formed on the polymerised strand of DNA (Figure 8). This method involves multiple rounds of a three-step cycle, specifically, synthesis of the particular DNA base on the single-stranded DNA template, then, fluorescence detection of the newly incorporated base and, finally, removal of the PCR reactants to allow the cycle to restart for the next base.

The final step of an NGS experiment is ‘data analysis’, which involves processing the large amount of short-read DNA reads that are generated (Figure 8) and is typically divided into two phases. The first phase involves taking the raw signals that the sequencing machine collects and processing them into ‘base calls’, which are assembled into sequencing reads (each with their associated quality scores). The second phase involves performing quality control on the reads, aligning them to a reference genome (see Section 3.6 below), and finally, the identification of variants using a variety of computational algorithms to detect the different genetic variations that may be present, ranging from the alteration of a single nucleotide to chromosomal alterations.

It is important to note that NGS analysis of the DNA extracted from FFPE tissues is not without its complexities. For example, formalin can induce various chemical modifications of the DNA that can introduce base substitution artefacts (‘false signals’, such as C>T/G>A, caused by cytosine deamination, and C>A/G>T, which mostly results from base oxidation) [8,9]. Formalin fixation can also result in information loss during the DNA sequencing process, such as due to an increased sequence duplication ratio (which increases the cost for unique coverage), DNA fragmentation, resulting in reduced library insert size, and decreased coverage uniformity [8,9]. Critically, on a background of non-uniform coverage and/or local areas of extremely low coverage, the artefacts observed in FFPE DNA may achieve such a high variant allele frequency (VAF) that they may ultimately be mistaken for biological variants, despite deep sequencing [77]. Nevertheless, FFPE-extracted nucleic acids are routinely used in genomic medicine in humans [7,9] as there are points within the multiple steps associated with the processing of the extracted nucleic acid in preparation for NGS that can be optimised to help reduce the artefacts [7,8]. In addition, there are computational algorithms that take into account the fact that the sequence from FFPE nucleic acids typically suffers from low-coverage regions, short insert sizes and various artefacts; various bioinformatic analyses can be applied to post-sequencing samples to ensure that high-quality, high-confidence results can be obtained [8,9].

The decision of whether to use WGS, WES or TGS for a study is weighed up by the relative advantages and disadvantages of each method (Table 2) and depends on the nature of the investigation. Many studies have used WGS, WES and/or TGS to characterise the genome of canine tumours. For example, WGS was used to validate copy number alterations found in canine glioma samples identified by microarray [78]. WGS has also been used to examine the mitochondrial DNA in a large number of transmissible venereal tumour samples from dogs (*n* = 449) [79]. Another study performed WGS analysis of canine OSA samples (*n* = 24) and identified recurrently mutated genes (*TP53*, *DMD* and *SETD2*) and focal chromosomal copy number (CN) alterations (CN gains spanning *PDGFRA* and *MYC*, and CN losses spanning *DMD*, *DLG2*, and *SETD2*) [80]. In another approach, WES was used to identify genetic variations that could potentially impact the chemotherapeutic response in dogs with multicentric B-cell lymphoma (*n* = 6) [81].

Other studies have used WES analysis to interrogate the genetics of canine tumours. For example, several groups have performed WES analyses of canine urinary bladder urothelial carcinomas (UCs) and identified *BRAF* as the most recurrently mutated gene, in particular, the hotpot V595E mutation, which was found in the majority of samples [24,82]. WES analysis of trio samples of canine primary OSA versus OSA metastasis versus normal tissue from the same dog (*n* = 10) showed that 4/10 (40%) metastases showed the acquisition of a likely pathogenic driver mutation that was not present in the primary tumour [80]. WES analysis of 28 canine cutaneous and subcutaneous mast cell tumours (MCTs) found 15 recurrently mutated genes, and the subsequent analysis of additional MCT samples by TGS (using a panel of 50 genes) identified recurrent mutations in *GNB1* (6/20, 30% cases), with Sanger sequencing of *GNB1* in further samples, determining an overall prevalence of *GNB1* mutations as 17.3% (14/81 cases), which is similar to the prevalence of the *KIT* alterations seen in these tumours [83]. WES analysis of canine mammary tumours (*n* = 183) found that *PIK3CA* was the most recurrently mutated gene (43% of tumours); many other genes in the PI3K-Akt pathway were also recurrently mutated, including *PTEN*, *PIK3R1* and *AKT1* [84]. WES analysis has also identified *TP53* and *KMT2D* as the most recurrently mutated genes in canine soft tissue sarcomas [85].

Finally, TGS has been used by many studies to understand the oncogenome of canine tumours. For example, several studies have used TGS (each employing panels with different numbers of cancer-associated genes) to characterise the genetic landscape of haemangiosarcoma (HSA) in canines, identifying recurrently mutated genes (such as *TP53*, *PIK3CA* and *LRP1B*), recurrent somatic copy number alterations and mutational signatures (including a UV signature in a subset of the cutaneous samples) [86,87,88,89]. Other studies have taken a pan-cancer approach and used targeted panels of 120–283 cancer genes to detect somatic mutations in various tumour types, with recurrently mutated genes being identified in over 90% of the clinical samples [2,90]. Similarly, another study using a targeted panel of cancer genes identified novel mutations with prognostic value across multiple cancer types and was able to demonstrate the benefit of genomically informed targeted treatment in dogs with cancer [3].

#### 3.5.2. NGS for Detecting Variations in the RNA

The most commonly used NGS method for detecting RNA variations is RNA sequencing (RNA-Seq). It can assess aspects such as gene expression profiles, alternative splicing events, allele-specific expression, and gene fusions. RNA-Seq provides several advantages over the traditional hybridisation-based (microarray) approaches as it can provide single-base resolution of transcriptional features, has a higher sensitivity for weakly expressed genes, has lower technical variation/higher levels of reproducibility and does not require prior knowledge of the genome of the organism.

The typical RNA-Seq workflow is very similar to that used for the analysis of DNA, with an extra step to generate a ‘cDNA library’ for each sample; specifically, the RNA extracted from the tissue is reverse-transcribed to cDNA. This is necessary as DNA is more stable, allows amplification (using DNA polymerases), and means that the sample can then be sequenced using DNA sequencing technologies (as per Section 3.5.1). RNA sequencing protocols can differ, depending on whether the fragmentation/size selection step is performed on the RNA or cDNA (or both), with each method having its upsides and downsides. However, as small RNAs are lost during the standard fragmentation/size selection step, appropriate size selection of the RNA is necessary to isolate the desired targets before the generation of the cDNA if miRNAs and non-coding RNAs are of interest.

Many studies have performed RNA-Seq on canine tumour samples, each using different tumour types and/or different applications. For example, RNA-Seq has been used to assess the expression values of genes within regions of CN gains/losses (as identified by WES); it identified a significant correlation between increased *MYC* expression in canine OSA samples showing somatic *MYC* CN gains (*p*  =  0.018) and low *SETD2* expression in those samples with somatic *SETD2* mutations [80]. RNA-Seq has also been used to identify fusion genes in canine cancers. For example, a study looking at three distinct human-comparable canine cancers found gene fusions (and breakpoints) that were present in both species, specifically, *IGK*::*CCND3* in B-cell lymphoma, *COL3A1*::*PDGFB* in dermatofibrosarcoma protuberans-like and *MPB*::*BRAF* in glioma [91]. Another RNA-Seq study found the presence of fusions that formed between *PDGFB* and various collagen genes in 10% of canine soft tissue sarcomas [85].

Mostly, RNA-Seq studies involve looking at differentially expressed genes (DEGs) between two groups. For example, a study performing RNA-Seq on canine urinary bladder UC (*n* = 11) and normal bladder samples (*n* = 3) found that the DEGs that were upregulated in the tumours were enriched for pathways involved in cell cycle-related processes, DNA repair and immune system processes, which led to the identification of a subset of UCs with an immunologically ‘hot’ tumour microenvironment (i.e., immune cell-infiltrated tumours) that may, thus, respond to therapy with checkpoint inhibitors [82]. RNA-Seq of canine mammary tumours (*n* = 157) identified three gene expression-based subtypes, one of which was transcriptionally similar to basal-like human breast cancer [84]. More recently, the gene expression profiles of canine multicentric high-grade B-cell lymphoma (*n* = 25) were assessed by RNA-Seq to compare cases where the subjects completed the chemotherapy protocol without relapse, versus those that relapsed during the treatment, and found that both changes in the tumour cells themselves and changes in the immune cell-tumour cell interactions were associated with chemotherapy efficacy [92]. Finally, other recent studies used RNA-Seq (adapted for small RNA sequencing) to analyse the differential expression of miRNAs in canine diffuse large B-cell lymphoma (6 samples) relative to controls (4 non-neoplastic samples) [93], and FFPE duodenal samples from canines with intestinal T-cell lymphoma (*n* = 9) relative to those with inflammatory bowel disease (*n* = 12) and to those with no/minimal inflammation [54].

#### 3.5.3. Methylation Sequencing

There are three basic approaches to whole genome DNA methylation profiling, specifically, affinity enrichment-based, restriction enzyme-based, and bisulfite conversion-based methods [94]. (1) Affinity enrichment-based methods use either antibodies (in the ‘methylated DNA immunoprecipitation’ (MeDIP-Seq) technique) or methylated-CpG binding proteins (in the ‘MBD-Seq’ technique) to capture and ‘pull down’ the methylated genomic regions. This allows the unmethylated genomic fraction to be washed away, after which the methylation-enriched portion can be sequenced. (2) Restriction enzymes-based methods utilise the ability of certain restriction enzymes (such as *Msp*I) to cleave the DNA at the site of methylation; therefore, 5-methylcytosine (5-mC) in gene promoters can be identified when the digested fragments are sent for sequencing. (3) In bisulfite conversion methods, the DNA is denatured and treated with sodium bisulfite, whereby any unmodified cytosines are converted to uracil but the methylated cytosine remains unchanged, which, thus, allows for the base resolution detection of cytosine methylation after sequencing of the bisulfite-treated DNA. Whole genome bisulfite sequencing (WGBS) and reduced-representation bisulfite sequencing (RRBS) are commonly used examples of the conversion method. The typical workflow is that the genomic DNA is extracted, and libraries are created. The libraries are then bisulfite-treated, PCR-amplified and sequenced. The only difference is that RRBS is a targeted DNA methylation profiling technique, as opposed to whole genome DNA methylation profiling. As such, it focuses on CpG-rich regions by using methylation-sensitive restriction enzymes to cleave unmethylated DNA at specific CpG sites with high GC density, followed by size selection of the fragmented DNA before bisulfite treatment.

RRBS analysis of canine mammary tumours (*n* = 2) and peripheral blood mononuclear cells (PBMCs; *n* = 2) found a number of differentially methylated regions in the tumours (relative to the PBMCs) in the promoter regions of various miRNAs, including some that have been reported in humans as cancer-associated miRNAs [95]. RRBS analysis of canine gliomas (*n* = 45) was used to classify them according to the methylation model used for human brain tumours and found that the majority of the canine samples were classed as paediatric gliomas [96]. More recently, RRBS analysis of canine OSA samples (*n* = 44) and human OSA samples (*n* = 24) found groups of highly correlated DNA methylation marks in both species and determined the underlying driver of differences in DNA methylation across the human and canine samples [97].

In addition to the modification of methylation status, epigenetic alterations can also involve the regulation of chromatin accessibility; the euchromatin (‘open’ chromatin) structure is permissible for access by DNA regulatory elements and, thus, transcription, whereas the heterochromatin (‘closed’ chromatin) structure is more compact and is not able to be accessed by the DNA elements needed for transcription. A technique to measure the accessibility of chromatin is the assay for transposase-accessible chromatin using sequencing (ATAC-Seq), first published in 2013 [98]. ATAC-Seq identifies accessible DNA regions using a hyperactive mutant Tn5 transposase that inserts sequencing adapters into open regions of the genome in a process known as ‘tagmentation’. First, a single-cell suspension is made from the tissue and the cells are lysed with a non-ionic detergent to yield pure nuclei. The chromatin is then fragmented and simultaneously tagmented to generate the library. After purification, the library is then PCR-amplified using barcoded primers and, finally, sequenced. The main advantage of this technique over the aforementioned ones is that it requires a relatively small number of input cells, has a reduced library preparation complexity and shorter hands-on time, and does not require a priori knowledge of the epigenetic mechanisms that exist in the tissue. A recent study employed a novel approach whereby both ATAC-Seq and RNA-Seq were performed on the primary OSA of a dog, to simultaneously capture the epigenomic and transcriptomic profiles within the same cell and to gain insights into the mechanisms driving intra-tumoural heterogeneity [99].

### 3.6. A Reference Genome for Domestic Dogs

Although there are different breeds of domestic dogs, they are all grouped into a single species, *Canis familiaris*. The genome of *Canis familiaris* contains ~2.4 giga base pairs. Annotation of the genome involves the process of identifying the functional elements in the sequences of a genome (such as genes) and attaching biological information to these elements. A high-quality, well-annotated genome assembly (i.e., a reference genome) is critical for both basic and clinical research into the genetics of an organism. Thanks to advances in both NGS technologies and analytical tools, assembling and annotating the genomic sequence of organisms is now readily achievable.

In 2005, the first high-quality draft genome sequence of the domestic dog was published, together with a dense map of SNPs that was used to reveal long-range haplotypes [100]. Genomic DNA from a purebred female Boxer (named ‘Tasha’) was sequenced using a whole-genome shotgun approach, providing 7.5× coverage, and assembled to create an initial assembly known as ‘CanFam1.0′ (followed by an updated assembly containing minor improvements, known as ‘CanFam2.0′) [100]. This breed was found to contain the least amount of genomic variation. However, as this draft genome was used to design multiple SNP arrays to perform genome-wide association studies to allow trait mapping (such as identifying the genes responsible for coat colour and morphology), the identification of mutations, such as those occurring in cancer, was not possible. This was due to the presence of ‘gaps’ in the draft genome, meaning a comprehensive annotated catalogue of genes, non-coding transcripts and regulatory elements was not available. In 2014, the ‘CanFam3.1′ genome assembly was released, which showed improved quality/completeness over the draft genome (by filling in many of the ‘gaps’ of CanFam2.0) and a new annotation was applied [101]. The assembly consists of 39 chromosomes and is composed of 20,257 coding genes, 10,081 non-coding genes and 613 pseudogenes [101]. This reference sequence has been widely used and has allowed the identification of variants (both single-nucleotide variants and copy number variants) in a large range of tumour types.

An accurate reference genome is critical for the variant calling (identification) process, as otherwise, ‘false’ variants may be called. These could arise from mis-mapping of the sequencing reads (such as Y chromosome sequence reads being incorrectly mapped to the autosomes; CanFam3.1 is from a female dog and so has no Y chromosome sequence) and/or gaps in the assembly (as sequencing reads that span these gaps would either map to the wrong position in the correct gene or map to an entirely incorrect gene) [102]. On this basis, it is worth mentioning that CanFam3.1 possesses over 20,000 gaps, with ~20% of them occurring within genes [103]. In September 2020, the Roslin Institute released the ‘ROS_Cfam_1.0′ assembly, created using the PacBio HiFi sequencing of genomic DNA from a male Labrador retriever. This breed was chosen as it is one of the world’s most popular pedigree breeds and has relatively high genetic diversity. In addition, being derived from a male dog, this assembly also includes data for the Y chromosome. The assembly consists of 40 chromosomes and is composed of 20,567 coding genes, 9944 non-coding genes and 610 pseudogenes. More recently, blood was taken from Tasha (the Boxer used to create CanFam1.0, 2.0 and 3.0), and long-read sequencing technologies were used to generate an improved, highly contiguous assembly, termed ‘Dog10K_Boxer_Tasha_1.0′, which closed > 23,000 of the gaps in the CanFam3.1 reference assembly and identified > 1200 new protein-coding transcripts [104]. Although the most widely used reference genome to date is CanFam3.1, this may change as there are now many more high-quality genomes from different dogs that have become available (Table 3).

It is important to note that the canine reference genome was generated from a single dog. In contrast, the latest human reference—a draft of the human pangenome—contains 47 assemblies from a cohort of genetically diverse individuals [105]. By containing the sequence and structural diversity found in different human populations, the pan-genome is more representative of our species. Of note, analysis of genomic data from 120 dogs of 60 different breeds revealed significant variation at a level much higher than that seen in the human population [106]. Thus, having a reference genome from a single dog is not representative of the canine species as a whole, i.e., a single genome cannot represent the genetic diversity of a species. Human genomic studies have identified tens of megabases within SVs that are polymorphic within the human population [107] and, due to the absence of these alternative alleles in the reference genome, more than two-thirds of SVs have been missed in studies that used short-read sequencing technologies (such as WES, TGS and short-read WGS) and the human reference assembly [108]. Thus, when sequencing a canine tumour and when there are SVs that are not present in the canine reference genome, that portion of the tumour genome will not be mapped and, thus, the structure of the sequence, the genes it contains and its effect on proximal gene regulation will remain unknown. Furthermore, in some cases, where there are closely related segmentally duplicated regions in a sequenced tumour, these can map back to a single locus in the reference genome and may cause erroneous somatic variant calls.

**Table 3:** Summary of the publicly available domestic dog genome assemblies. Shown here are the name of the assembly, the breed and sex of the dog that was sequenced (as well as the name of the individual dog, if known) and the accession ID for the assembly, which is available on GenBank (https://www.ncbi.nlm.nih.gov/genbank/; accessed on 16 December 2023). Abbreviations: F, female; M, male; TRI, The Roslin Institute.

Assembly Name	Breed (Sex); Name	Accession ID	Ref
CanFam3.1	Boxer (F); ‘Tasha’	GCA_000002285.2	[101]
ROS_Cfam_1.0	Labrador Retriever (M)	GCA_014441545.1	TRI, 2020
ASM864105v3 (synonym: CanFam_GSD)	German Shepherd (F); ‘Nala’	GCA_008641055.3	[109]
ASM1204501v1	Labrador Retriever (M); ‘Yella’	GCA_012045015.1	[110]
CanFam_Bas	Basenji (F); ‘China’ Basenji (M); ‘Wags’	GCA_013276365.1 GCA_013276365.2	[111]
UU_Cfam_GSD_1.0 (synonym: CanFam4)	German Shepherd (F); ‘Mischka’	GCA_011100685.1	[103]
UMICH_Zoey_3.1 (Synonym: CanFam5)	Great Dane (F); ‘Zoey’	GCA_005444595.1	[112]
Dog10K_Boxer_Tasha_1.0 (synonym: CanFam6)	‘Tasha’ (details as above)	GCA_000002285.4	[104]
CA611_1.0	Cairn Terrier (M)	GCA_031010295.1	[113]
BD_1.0	Bernese Mountain Dog (F)	GCA_031010765.1	[113]
OD_1.0	Bernese Mountain Dog (M)	GCA_031010635.1	[113]

### 3.7. Germline Databases

Some WGS, WES or TGS studies performed on canine tumour samples have also sequenced matched normal tissues from the same dogs and have, thus, been able to identify germline variants representing potential tumour susceptibility variants. For example, a study that performed WGS and WES sequencing of healthy tissue samples from dogs with osteosarcoma (OSA) looked for coding germline variants (with a predicted impact on the protein of ‘high’ or ‘moderate’) in 28 genes that were previously associated with both canine and human OSA and found recurrent variants in *APC2*, *BLM*, *BRCA2*, *TP53*, *RB1*, *WRN* and *CDKN2B* [80]. Germline variants were also identified in *SETD2* (1 sample) and *DMD* (2 samples); however, the significance of these alterations is unknown [80].

However, most canine NGS studies only sequence the tumour sample and not ‘matched’ normal tissue from the same dog (such as blood or healthy tissue). This may be for a variety of reasons, including a lack of availability (if the tumour was sampled for diagnostic purposes, then blood or other healthy tissues may have not needed to be collected) and/or financial resources (the sequencing of a matched normal sample for every tumour sample doubles the cost of the study). However, as well as not being able to identify germline mutations (and, thus, identify potential cancer predisposition genes), the consequence is the inability to filter out germline single-nucleotide polymorphisms (SNPs) from the tumour sequencing data. Thus, a proportion of the somatic variants reported will be germline variants. Unlike humans, where there have been significant efforts in sequencing normal tissue to identify germline variants, such as the UK10K project [114], the same project does not exist for dogs. However, progress is starting to be made in this direction. The National Human Genome Research Institute (NHGRI) Dog Genome Project, which focuses on the genetics of health and body structure in the domestic dog, performed WGS analysis on normal tissue from 722 canines (including 526 purebred dogs, 142 random-bred dogs and 54 wild canids) and identified over 91 million nucleotide variants, creating an extensive catalogue of canine genomic variation [115]. More recently, the international Dog10K project, which aims to sequence and analyse several thousand canine genomes, performed WGS analysis on normal tissue from 1987 canines (including 1611 dogs from 321 breeds, 309 village dogs, 63 wolves and 4 coyotes) and identified 34 million single-nucleotide variants (SNVs) and 144,000 structural variants (deletions, insertions, duplications and inversions ≥ 50 bp in size), making the Dog10K reference panel the largest canine dataset assembled to date [116]. This will be an invaluable resource for the field of canine oncogenomics.

## 4. Emerging Fields for Genetic Investigations of Canine Tumours

### 4.1. RNA Analysis

It is important to note that microarray and RNA-Seq techniques analyse the expression of RNAs from large populations of cells (i.e., ‘bulk transcriptomics’); thus, information about the heterogeneity of the tumour at a cellular level is not captured. To that end, new methods have been developed to allow the sequencing of RNA from individual cells and to know the precise location of that cell within the tumour mass.

#### 4.1.1. Single-Cell RNA Sequencing (scRNA-Seq)

scRNA-seq provides a transcriptional profile of individual cells with high resolution; the major use of this technique has been in the assessment of heterogeneity by comparison of transcriptional similarities and differences within a population of cells (such as tumours, immune cells, etc.). Another use has been the identification of rare cell populations that would not be detected in pools of cells, such as the different populations of malignant cells within a tumour mass [117]. scRNA-seq can also trace lineages and developmental relationships between heterogeneous cellular states within tumours [118]. Approximately 20 different scRNA-seq protocols have been published to date, the key differences being in the length of the transcript data they provide [119]. However, the typical scRNA-Seq workflow is very similar to that used for RNA-Seq, with the addition of an initial step that focuses on isolating single cells from the tissue samples. This can be achieved through a variety of different techniques, including microdissection and manipulation, cell sorting on a flow cytometry platform, microfluidic platforms, and droplet-based methods. The RNA is then extracted from the single cells, and the protocol then essentially follows that for RNA-Seq, with the barcoding/indexing of cDNA libraries from individual cells allowing for pooling of the cDNA libraries before sequencing. However, in comparison to RNA-Seq, the data from scRNA-Seq experiments are relatively sparse and have a higher level of technical ‘noise’ due to a high frequency of ‘dropout events’ (which is when there is a lack of detection of specific transcripts, i.e., ‘zero observations’) and, as such, the numbers of expressed genes detected from single cells are typically lower compared with those from the bulk population. From the scRNA-Seq data, patterns of gene expression can be identified through gene clustering analyses, which involve grouping the cells into subpopulations (‘clusters’), whereby those in a particular cluster are more similar to each other than to those in other clusters (in terms of marker gene expression and abundance).

The first scRNA-Seq study was published in 2009 and used mouse cells [120]. Since then, the technique has proved very powerful in human oncology for teasing apart complex biological processes and gaining insight into cellular heterogeneity and diversity. In veterinary oncology, only a few scRNA-Seq studies have been performed on canine tumours to date. The initial trialling of scRNA-Seq analysis was performed on canine urinary bladder UC and focussed on the immune cell population (CD45+ cells) to demonstrate how this technique can be used to identify a novel role for genes in this tumour type; *GPR183* showed increased expression in cells of a particular cluster, and whilst this gene has a known role in the immune system, its role in canine UC has not yet been investigated [121]. Another study used the scRNA-Seq of two cell lines derived from canine OSA to demonstrate significant intratumoral heterogeneity in gene transcription expression patterns within the cell lines [122]. More recently, a study used scRNA-Seq to characterise the transcriptional heterogeneity of circulating leukocytes from dogs with OSA (*n* = 10) versus healthy dogs (*n* = 7), and used the data to investigate how the presence of OSA impacts leukocyte transcriptional programs; the relative proportion of PMN-MDSCs and M-MDSCs were increased in dogs with OS [123]. The same group also used scRNA-Seq to characterise the tumour microenvironment of dogs with OSA (*n* = 6) in terms of its cellular and molecular composition; the analysis of 35,310 cells revealed the presence of 41 transcriptomically distinct cell types, including 30 distinct immune cell types, 9 unique tumour populations, 1 cluster of fibroblasts, and 1 cluster of endothelial cells [124]. Following the successful findings reported in these studies, it is anticipated that scRNA-Seq will become a more widely used tool in characterising canine tumours.

#### 4.1.2. Spatial Transcriptomics (ST)

ST provides spatial resolution of the transcriptional activity within intact tissue, either for single cells or regions of cells, and has an advantage over RNA-Seq and scRNA-Seq in that it removes the need for tissue dissociation and preserves the spatial context of cells. ST encompasses three main technologies, specifically, in situ hybridization (ISH), in situ sequencing (ISS), and in situ capturing (ISC)-based technologies (each is detailed below and reviewed in [125]). These three technologies can be broadly divided into either targeted approaches, for which specific genes are interrogated (ISH and ISS), or unbiased approaches for transcriptome-wide profiling (ISC). Each method comes with advantages and disadvantages, as well as differences in the degree of resolution (i.e., how precise a location is retained for each transcript). Targeted approaches show higher efficiency (detecting a higher percentage of transcripts within the tissue), but fewer unique genes can be assayed at one time (100–10,000 genes, depending on the method used). In contrast, unbiased profiling can capture the whole transcriptome, but at lower efficiency (i.e., only a fraction of RNA copies for each gene is captured) [126].

(1) ISH methods (such as seqFISH, merFISH and seqFISH+) detect specific target genes through the use of fluorescently labelled probes that are complementary to the RNA transcript of interest, with the signals from the probes providing quantitative determination of the transcripts in that spatial context.
(2) ISS methods (such as Padlock Probe ISS and FISSEQ) involve fixation of the mRNA, followed by in situ reverse transcription to form cDNA. Padlock probes (PLPs; single-stranded DNA probes designed against targets of interest) are then hybridised with the tissue section and allowed to bind with the cDNA. Bound PLPs are amplified by a process known as rolling circle amplification (RCA) and are labelled with fluorophore-conjugated probes, which allow their detection. ISS can detect up to a few hundred genes per sample and wide-field imaging enables high throughput.
(3) ISC methods (such as 10× Genomics Visium, Slide-seq and Seq-Scope) capture transcripts in situ, whilst NGS is performed ex situ and, as such, enables unbiased capture of the entire transcriptome. The general ISC strategy uses slides with arrays of ‘capture spots’ consisting of barcoded reverse transcription (RT) primers with poly-T sequences that capture the mRNA transcripts. Tissues are sectioned onto these slides to allow hybridisation of the transcripts to the spots, after which RT is performed, and the resulting cDNAs are extracted for NGS. After sequencing, the reads are superimposed onto the tissue image using the positional barcodes, thus allowing spatial visualisation of the transcriptome.

In human oncology studies, ST has been widely used in characterising the spatiotemporal heterogeneity of cancers [127] and the tumour microenvironment [128]. For example, a recent study performed integrative single-cell and spatial transcriptomic analysis of HPV-negative oral squamous cell carcinomas to comprehensively characterise the transcriptional landscape of malignant cells in the centre of the tumour mass, versus those at the leading edge of the mass, and found correlations with the transcriptional program at each of these sites and outcome/prognosis [129]. In veterinary oncology, there have only been a few studies that have used ST technologies to date. Examples include the use of a commercially available ISH assay for the detection of transcripts in FFPE tissue (RNAScope^TM^) to evaluate *KIT* mRNA in canine mast cell tumours (*n* = 60), finding a statistically significant correlation between *KIT* mRNA expression and histological grade [130]. Another commercially available ST product is the GeoMx^®^ digital spatial profiler (DSP); this can be used with the Canine Cancer Atlas (CCA) panel, which profiles 1962 RNA targets with spatial resolution and is designed for transcripts specific to the onset and progression of cancer in dogs (with the targets including those involved in common canine cancers such as lymphoma, OSA, UC, melanoma and glioma), as well as the TME and immune response. Recent use of the GeoMx^®^ DSP with the CCA panel demonstrated the specificity and sensitivity of ST across a wide variety of tissues (FFPE sections from canine OSA, HSA and glioma, as well as normal colon, brain and lymph node samples from canines) [131]. Thus, it is anticipated that more studies using ST will soon follow.

### 4.2. Prediction of Genetic Alterations from Histology Slides

The correlation between the genotype and histologic tumour phenotype (assessed by haematoxylin and eosin (HE)-stained slides) is a well-known phenomenon that was first described in humans in the 1980s at the end of the last century, with the demonstration that mutation of the oncogene *v-fps* was associated with changes in histologic tumour growth pattern, malignancy and stroma composition [132]. With the recent developments in computational pathology, the histologic genotype-phenotype correlation has gained much attention, mainly because of the power of deep learning approaches in analysing high numbers of histology specimens and their ability to detect even subtle and yet undefined histopathologic features [133,134]. When applying deep learning approaches, the network uses specific features to identify genetic alterations [135,136]. For most studies, these features are non-interpretable by a pathologist [137]. However, some human-interpretable features have been identified for selected cancer types or specific genetic mutations (i.e., specific histopathologic parameters such as nuclear size, mitosis and tumour morphology), and are associated with defined molecular alterations [138,139,140]. Given that interpretable features are quantifiable, there is the potential to define thresholds for a defined histologic feature to predict a specific genotype on a tissue or even at the single-cell level.

**Figure 9:**
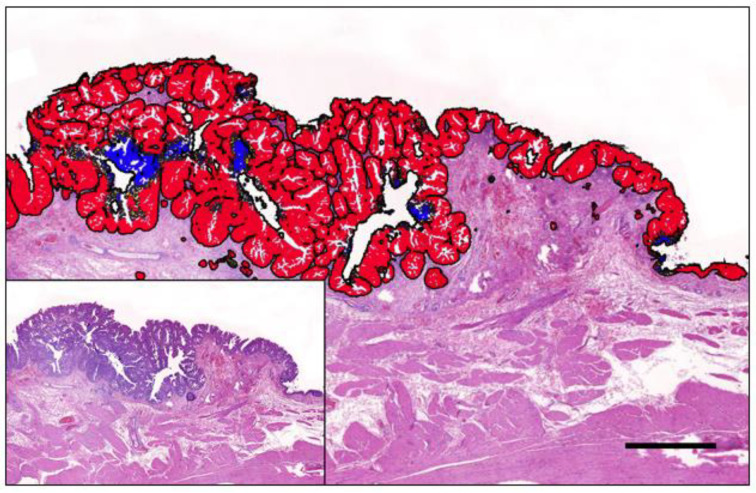
Microphotograph of a HE-stained canine urothelial carcinoma tissue section. AI histology correctly predicted this case to be positive for the *BRAF* p.V595E mutation, as confirmed by PCR. AI labelling—red: BRAF positive; blue: BRAF negative; yellow: uncertain. The bar indicates 1 mm. Inset: the same section without labelling.

Initial studies in humans demonstrated such genotype predictability in specific cancer types and for only a few individual genes [141,142]. However, more recently, genotype–phenotype correlation investigations have been performed as pan-cancer studies wherein an extensive range of genetic alterations are considered [133,134,143]. In veterinary oncology, investigations of deep learning-based tumour histology are starting to be performed on canine tumours, wherein artificial intelligence (AI)-based methods are using digitized whole-slide images (WSIs) to make pathological diagnoses, such as diagnosing canine skin tumours (of seven different types) [144,145] and canine osteosarcoma (where the AI program was able to identify specific histologic subtypes that may have prognostic value) [146]. However, to date, there has only been one genotype–phenotype correlation study in canines; it was recently shown that deep learning using a commercial AI histology software could detect the *BRAF* p.V595E mutation on HE-stained canine bladder cancer tissue sections (Figure 9), with a sensitivity of 89% [147].

Undoubtedly, we expect more veterinary genotype–phenotype correlation reports to be available in the following years. However, the basis for this is, of course, that the genetic signatures of the canine tumours must first be known before a histomorphological correlation can be made. Furthermore, regarding the clinical implications of such AI tools, thorough validation is required to guarantee the accuracy and robustness of a given image-analysis program [148,149]. Clinical AI systems need to be safe, and the European Council very recently defined an artificial intelligence act to ensure this in the medical and other fields. For selected biomarkers, rigorous evaluation has already been performed and AI algorithms have been approved and are available for diagnostic use [150,151,152]. For newly identified markers, clinical translation is dependent on validation studies, which may delay the implementation of these AI biomarker detection systems.

## 5. Important Considerations

### 5.1. Adaptation of Technologies Used in Human Research

A critical point to consider is that these molecular technology methods have been primarily ‘adapted’ from their initial use in human oncology research and diagnostics. In this respect, the ‘older’ methods have had the benefit of time: time for multiple independent assessments of the accuracy of the technique and validation of its adaption for use in veterinary oncology. The newer methods that may be used ‘out of the box’ from their uses in human oncology need to come with warnings that validation is first required. In addition, it is essential that researchers, diagnosticians and clinicians are aware of the possibilities and limitations of the various molecular methods in order to plan studies correctly and to present and interpret the results correctly.

### 5.2. Generation of Databases for Our Genomic Knowledge

There are many publicly accessible databases that curate, collate and make available the huge amount of genomic data generated from sequencing studies that have been performed on human tumours. For example:

- *Cancer Genome Atlas (TCGA)*: collected, characterised, and analysed human cancer samples from >11,000 patients over a 12-year period (https://www.cancer.gov/ccg/research/genome-sequencing/tcga)
- *Catalogue Of Somatic Mutations In Cancer (COSMIC)*: the world’s largest and most comprehensive resource for exploring the impact of somatic mutations in human cancer (https://cancer.sanger.ac.uk/cosmic)
- *cBioPortal for Cancer Genomics*: provides visualisation, analysis and a download of large-scale human cancer genomics data sets (https://www.cbioportal.org/)

We need similar harmonisation and archiving of the data coming from the increasing number of canine cancer genomic studies, with relevant clinical data if possible, and need the resulting databases to be made publicly available. As has been demonstrated in human medicine, these approaches can expedite our understanding of the genomic alterations in canine cancers and will provide a springboard for the development of targeted therapies for veterinary medicine.

### 5.3. Ethics

As is the case in human studies, it is important to consider the ethics of performing molecular analyses on tumour and germline samples from dogs. The vast majority of canine molecular characterisation studies performed to date have involved the analysis of spontaneously occurring neoplasms from pet dogs, rather than neoplasms arising in carcinogen-exposed laboratory/research dogs. Typically, canine oncology genomic research is performed on samples that have been collected for diagnostic purposes (either on a live dog or during a necropsy) or for treatment purposes (such as the surgical excision of a mass). Samples may be a portion of the tumour (either in an FFPE block or FF) or as liquid biopsies, such as urine or blood, for example. In all cases, the owner consents to the tissue/liquid biopsy sample also being used for research (or teaching) purposes, including the generation of genetic data from the sample. Indeed, as most dog owners expect more veterinary diagnostic and treatment options to be available and are willing to spend increasing amounts of money on expensive life-saving treatments for their beloved pets [153], they are willing to let their dog’s sample be used for research as they understand the benefits it can bring by allowing a better understanding of the disease and the hope of generating better diagnostic, prognostic and/or therapeutic options for other dogs in the future.

## 6. Conclusions

There is a plethora of techniques to use for genomic analysis of canine cancers (summarised in Table 4). Some methods have been around for many decades, but newer methods are becoming available due to technological advances. However, both have a place in canine oncogenomics, as the method of choice depends on many factors:

(1) *The nature of the investigation*; for example, does it require analysing the whole genome or just a portion of the genome, an entire gene or just a specific portion of a gene? Is single-cell and/or spatial analysis required, or will bulk analysis of the tumour suffice?
(2) *Availability of access to the relevant equipment required*; for example, does the method need NGS technology or will a PCR machine suffice?
(3) *Complexity of the data output*; for example, does it require a bioinformatician to analyse the sequencing data, or does it not require any (or only minimal) computational analysis?
(4) *Time frame*; for example, an NGS and the subsequent data analysis can take several months.
(5) *Cost*; for example, WGS costs more than WES, and RNA-Seq costs more than qPCR.

Thus, with the availability of so many molecular technologies, the future is looking bright for the field of canine oncogenomics; undoubtedly, we will soon see the benefits of this in veterinary clinics, with the increasing identification of diagnostic markers, prognostic markers and therapeutic targets.

## Figures and Tables

**Table 1:** Overview of the most common sequencing platforms. Abbreviations: TGS, targeted sequencing; WES, whole exome sequencing; WGS, whole genome sequencing.

Platform	Sequencing Technology	Considerations
Illumina	Uses a sequencing-by-synthesis approach whereby DNA fragments immobilised on a flow cell are amplified into clonal clusters, and the fluorescent signal released during the incorporation of the fluorescently tagged nucleotides into the growing DNA strand is translated into a base call	Short read lengths (up to 600 bases) High accuracy High throughput Suitable for a wide range of applications: WGS, WES, TGS, RNA-Seq and more
Ion Torrent	Uses a sequencing-by-synthesis approach whereby hydrogen ions released during the incorporation of a nucleotide into the growing DNA strand are detected and translated into a base call	Short read lengths (up to 400 bases) Simplified sequencing procedure yielding reduced costs Suitable for TGS, small genome sequencing and RNA-Seq
Oxford Nanopore Technologies	Uses nanopore sequencing, whereby the change in electrical current made by a single DNA molecule passing through a nanopore is detected and translated into a base call	Extremely long read lengths (up to 2 million bases) Higher error rate than Illumina or Ion Torrent Suitable for SV detection and de novo genome assembly
Pacific Biosciences (‘PacBio’)	Uses single-molecule real-time (SMRT) sequencing, which allows for the sequencing of individual DNA molecules in real time	Very long read lengths (up to 20,000 bases) Higher error rate than Illumina or Ion Torrent Suitable for SV detection and complex genomic regions

**Table 2:** Comparison of the advantages and disadvantages of whole genome sequencing (WGS), whole exome sequencing (WES) and targeted sequencing (TGS).

Method	Advantages	Disadvantages
WGS	Detects coding and non-coding variants. Detects structural variants.	High cost. Huge volume of data to store and analyse.
WES	Detects only the coding regions (exome), thus reducing cost as it only sequences a small portion of the genome (1–2%); the majority of disease-causing variants are found in the exome. Faster than WGS as there is less data to be analysed.	Potential disease-causing variants outside the exome will be missed. Copy number alterations can be detected, but potentially less accurately than WGS. Capture-based methods require greater coverage/deeper sequencing at each locus to achieve a sensitivity comparable to WGS, due to capture bias for different regions and coverage loss because of off-target binding.
TGS	The high depth of coverage allows the detection and quantification of rare and low-frequency variants. Faster than WGS, as there is less data to be analysed, and cheaper than WGS as fewer regions of the genome need to be sequenced.	Potential disease-causing variants outside the targeted region will be missed. Copy number alterations can be potentially detected, but it depends on the number of genes being targeted and is much less accurate than WGS. Capture-based methods require greater coverage/deeper sequencing at each locus (as per WES).

**Table 4:** A summary of the different types of molecular analyses available to characterise the genome of canine cancers.

Molecular Technology		Method	Use	Comments
PCR	PCR (DNA), RT-PCR (RNA)	Amplifying specific regions of DNA/RNA	Amplification of region of interest for further analyses, such as quantification or sequencing	Fast and cheap
	qPCR (DNA), RT-qPCR (RNA)	Real-time detection and quantification of specific DNA/RNA regions by fluorescence intensity	qPCR: detection of SNVs or small indels RT-qPCR: gene expression profiling	Fast and cheap
	dPCR (DNA, RNA)	Absolute quantification of specific DNA/RNA regions by fluorescence signal in droplets	dPCR: mutation analysis dPCR with RT-PCR: mRNA and miRNA expression quantification	Higher sensitivity than qPCR
DNA microarray	Gene expression	Nucleic acid fragments labelled with a fluorescence dye by PCR or RT-PCR on a solid surface	Measurement of thousands of RNA transcripts in a single experiment	High throughput
	aCGH	Quantitatively compares the fluorescence signal intensity from test DNA and control DNA	Detection of CNVs	Cheaper than NGS
Nuclease protection assay	qNPA	After hybridisation with a probe, the targeted transcript is transferred to an array plate	Detection and quantification of mRNA expression	Highly suited for FFPE samples
Sanger sequencing		PCR including fluorophore-labelled nucleotides and capillary gel electrophoresis of products	Determines the DNA sequence of individual exons or genes	Fast, cheap
NGS (DNA)	WGS	Creating a library of the sample by PCR with molecular barcodes, selection of regions of interest for WES or TGS by RNA capture probes ‘baits’, then clonal amplification by PCR, library sequencing and data analysis	Sequencing all nucleotides of the genome including chromosomal and mitochondrial DNA	See Table 2
	WES		Sequencing the entire coding region (i.e., all the exons)	
	TGS		Sequencing a selected portion of the genome (i.e., genes of interest)	
NGS (RNA)	RNA-Seq	As NGS for DNA, but with an additional step for creating cDNA	Gene expression profiles, alternative splicing events, allele-specific expression and gene fusions	High sensitivity and reproducibility
Methylation sequencing	RBBS, ATAC-Seq, Microarray-based methylation profiling	Different techniques to determine the methylation of cytosines and ‘open’ regions of chromatin	Analysing the methylation profile and chromatin accessibility	For epigenetic investigations
Spatial transcriptomics	ISH-, ISS- and ISC-based methods	In situ mRNA investigation by FISH or sequencing	Transcriptomics in situ	Investigating the cell origin of an mRNA profile

## Data Availability

No new data were created.
